# Induction of a Stringent Metabolic Response in Intracellular Stages of *Leishmania mexicana* Leads to Increased Dependence on Mitochondrial Metabolism

**DOI:** 10.1371/journal.ppat.1003888

**Published:** 2014-01-23

**Authors:** Eleanor C. Saunders, William W. Ng, Joachim Kloehn, Jennifer M. Chambers, Milica Ng, Malcolm J. McConville

**Affiliations:** Department of Biochemistry and Molecular Biology, Bio21 Institute of Molecular Science and Biotechnology, University of Melbourne, Parkville, Victoria, Australia; University of Iowa, United States of America

## Abstract

*Leishmania* parasites alternate between extracellular promastigote stages in the insect vector and an obligate intracellular amastigote stage that proliferates within the phagolysosomal compartment of macrophages in the mammalian host. Most enzymes involved in *Leishmania* central carbon metabolism are constitutively expressed and stage-specific changes in energy metabolism remain poorly defined. Using ^13^C-stable isotope resolved metabolomics and ^2^H_2_O labelling, we show that amastigote differentiation is associated with reduction in growth rate and induction of a distinct stringent metabolic state. This state is characterized by a global decrease in the uptake and utilization of glucose and amino acids, a reduced secretion of organic acids and increased fatty acid β-oxidation. Isotopomer analysis showed that catabolism of hexose and fatty acids provide C4 dicarboxylic acids (succinate/malate) and acetyl-CoA for the synthesis of glutamate via a compartmentalized mitochondrial tricarboxylic acid (TCA) cycle. *In vitro* cultivated and intracellular amastigotes are acutely sensitive to inhibitors of mitochondrial aconitase and glutamine synthetase, indicating that these anabolic pathways are essential for intracellular growth and virulence. Lesion-derived amastigotes exhibit a similar metabolism to *in vitro* differentiated amastigotes, indicating that this stringent response is coupled to differentiation signals rather than exogenous nutrient levels. Induction of a stringent metabolic response may facilitate amastigote survival in a nutrient-poor intracellular niche and underlie the increased dependence of this stage on hexose and mitochondrial metabolism.

## Author Summary

*Leishmania* are sandfly-transmitted parasitic protozoa that cause a spectrum of important diseases in humans. While the core metabolism of the readily cultivated insect (promastigote) stage has been studied, much less is known about the metabolism of the obligate intracellular amastigote stage, which proliferates within the mature lysosome of mammalian macrophages and is the target of anti-parasite therapies. We have used ^13^C-tracing experiments to delineate the major pathways of carbon metabolism in different promastigote stages, as well as amastigote stages generated in culture and isolated from animal lesions. Both dividing and non-dividing promastigotes exhibited high metabolic activity, with excessive rates of glucose and amino acid consumption and secretion of metabolic end-products. In contrast, both amastigote stages exhibited a stringent metabolic phenotype, characterized by low levels of glucose and amino acid uptake and catabolism and increased catabolism of fatty acids. This phenotype was not induced by nutrient limitation, but is hard-wired into amastigote differentiation. This response may lead to increased dependence on hexose catabolism for anabolic pathways, as chemical inhibition of *de novo* glutamate and glutamine biosynthesis inhibited parasite growth in macrophages. This study highlights key aspects of amastigote metabolism that underpin their capacity to survive in macrophage phagolysosomes.

## Introduction

Kinetoplastid parasites, belonging to the genus *Leishmania*, are responsible for a spectrum of diseases that affect over 12 million people worldwide, ranging from self-limiting cutaneous infections to disseminating mucocutaneous and lethal visceral infections [1], [2](http://www.who.int/leishmaniasis/en/). Infection of the mammalian host is initiated by flagellated promastigote stages that develop within the digestive tract of the sand fly vector. Following an initial proliferative phase, promastigotes differentiate to non-dividing metacyclic stages that are the primary stage injected into the skin during a sand fly bite. Metacyclic promastigotes are phagocytosed by macrophages, either directly or after initial uptake by neutrophils, and are delivered to the mature phagolysosome compartment where they differentiate to non-motile amastigote stages [2]. Amastigotes are responsible for both acute as well as long-term latent infections that can lead to reactivation of disease years or decades after the primary infection, particularly in immunocompromised individuals [2]. How these stages survive within the macrophage's mature phagolysosome remains poorly defined [3].

Intracellular pathogens, such as *Leishmania*, are dependent on the salvage of essential nutrients and carbon sources from their host cell for growth with the metabolic pathways involved in their utilization being crucial determinants of virulence [4], [5]. The macrophage phagolysosome is expected to have a complex nutrient composition as a result of the constitutive internalization of macromolecules and their degradation by lysosomal proteases, lipases and glycosidases, although the steady state levels of some carbon sources, such as sugars, may be limiting [6], [7]. Intriguingly, *Leishmania* constitutively express most enzymes involved in central carbon metabolism, including enzymes required for the catabolism of glucose, amino acids and fatty acids [8]–[11]. While stage-specific changes in both the level of expression and post-translational modification of a number of proteins in central carbon metabolism have been observed [10]–[13], these are generally modest compared to those seen in other eukaryotic or prokaryotic pathogens and the physiological significance of altered enzyme expression levels remain poorly defined. These studies suggest that both promastigote and amastigote stages of *Leishmania* have a broadly similar metabolic potential, raising questions as to how and to what extent *Leishmania* metabolism is regulated during differentiation and/or in response to nutrient levels in different host niches.

A number of early studies, as well as more recent studies using comprehensive ^13^C-stable isotope resolved metabolomics, have led to a detailed dissection of carbon metabolism of *Leishmania* promastigote stages. In common with some other trypanosomatid stages [14]–[16], *Leishmania* promastigotes preferentially catabolize sugars via an atypically compartmentalized glycolytic pathway, in which the first five enzymes are located in modified peroxisomes termed glycosomes [17]. The ATP and NAD consumed in these early glycolytic reactions are regenerated, at least in part, by fermentation of phosphoenolpyruvate (PEP) to succinate. The end-products of glycolysis and succinate fermentation can be further catabolized in a canonical tricarboxylic acid (TCA) cycle, promastigotes to generate reducing equivalents and anabolic precursors [18]. A striking feature of promastigote metabolism is the apparent lack of feedback regulation of glycolytic fluxes, with glucose uptake in standard culture medium generally exceeding the capacity of mitochondrial metabolism to completely oxidize internalized glucose to CO_2_, leading to profligate secretion of partially catabolized intermediates such as succinate, acetate and alanine [19]. While *Leishmania* amastigotes are thought to express a similar repertoire of enzymes as promastigotes, early biochemical studies showed that amastigote differentiation was associated with marked changes in carbon source utilization [20]. In particular, amastigotes of several *Leishmania* species were found to exhibit reduced glucose uptake, as compared to rapidly dividing promastigotes, while simultaneously increasing the uptake of amino and fatty acids [20]. However, the extent to which amino and fatty acids were catabolized by canonical energy generating pathways was not defined in these studies. Moreover, subsequent genetic studies indicated that *Leishmania* amastigotes are highly dependent on hexose uptake and catabolism *in vitro* and *in vivo* despite having reduced capacity to take up sugars. Specifically, a *L. mexicana* mutant lacking the major hexose transporters, exhibited increased sensitivity to elevated temperature and oxidative stress when cultivated *in vitro* and was unable to proliferate in macrophages [21], [22]. Similarly, a *L. major* mutant with a defect in amino sugar catabolism was highly attenuated in both *ex vivo* macrophage and mouse models of infection [23]. It remains to be determined whether the dependence of amastigotes on hexose metabolism reflects the role of down-stream pathways in energy metabolism and/or production of essential anabolic precursors.

In the current study, we have used comprehensive ^13^C-stable isotope resolved metabolomics, previously applied to rapidly dividing promastigote stages [18], to investigate the carbon metabolism of non-dividing promastigotes and both *in vitro* differentiated and lesion-derived amastigotes. We find that both dividing and non-dividing promastigote exhibit a similar glucose-wasting metabolism despite their differences in growth rate. In contrast, both *in vitro* differentiated amastigotes and lesion-derived amastigotes exhibit a distinctive glucose-sparing metabolism in which the rate of glucose and amino acid consumption and the release of metabolic end-products is strongly decreased. The development of this stringent metabolic response was associated with increased rates of fatty acid β-oxidation and the operation of a compartmentalized TCA cycle configured for the anabolic production of α-ketoglutarate for glutamate/glutamine synthesis. Chemical inhibition of either the TCA cycle or glutamine synthetase strongly inhibited amastigote growth and viability *in vitro* and in infected macrophages, indicating that glucose and fatty acid metabolism is essential for sustaining amino acid synthesis. These studies demonstrate that pronounced stage-specific changes in *Leishmania* central carbon metabolism occur, despite the absence of transcriptional regulation, and that the stringent metabolic response of amastigotes may confer protection against elevated temperature and oxidative stresses encountered in the mammalian host.

## Results

### *In vitro* differentiated amastigotes enter a semi-quiescent state characterized by a glucose-sparing metabolism

**Figure 1:**
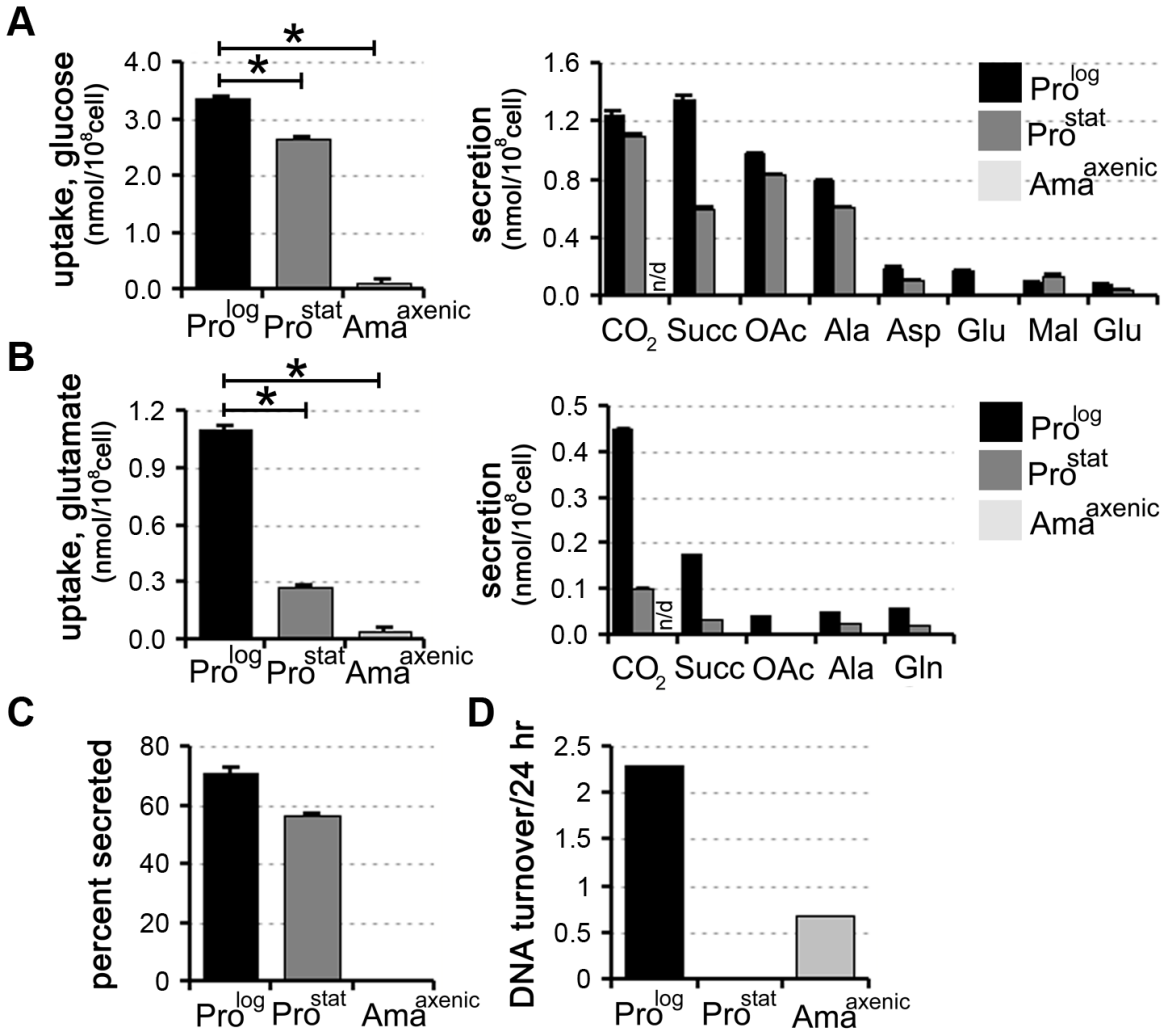
Carbon source utilization by different *L. mexicana* developmental stages. L. mexicana Pro^log^, Pro^stat^ and Ama^axenic^ were suspended in chemically defined medium (CDM) containing ^13^C-U-glucose (A) or ^13^C-glutamate (B) and the rate of utilization of the proffered ^13^C-carbon source (left hand panel) and production of overflow metabolites (right hand panel) assessed by ^13^C-NMR analysis of the conditioned medium. ^13^CO_2_ (detected as H^13^CO_3_^−^) was only quantified in the medium of promastigote stages, as it is not retained in the acidified amastigote medium. C. Proportion of internalized ^13^C-glucose that was secreted as partially oxidized end-products by Pro^log^, Pro^stat^ and Ama^axenic^. Similar relative levels of secretion were observed in the two promastigote stages (∼50%), while negligible secretion was observed in Ama^axenic^. D. Replication rates of different L. mexicana developmental stages. Pro^log^, Pro^stat^ and Ama^axenic^ were cultivated in RPMI supplemented with D_2_O (5% v/v, final) and the rate of DNA turnover determined by kinetic analysis of deuterium incorporation into adenosine deoxyribose. All data represent mean (n = 3) and standard deviation and are representative of several independent experiments.

We have recently shown that rapidly dividing *L. mexicana* promastigotes (Pro^log^) co-utilize glucose and selected non-essential amino acids when cultivated in completely defined medium (CDM) containing a range of carbon sources [18]. To assess the extent and rate at which other developmental stages utilize these carbon sources, stationary phase *L. mexicana* promastigotes (Pro^Stat^) and *in vitro* differentiated amastigotes (Ama^axenic^) were suspended in CDM medium containing either ^13^C-U-glucose or ^13^C-glutamate and the rate of utilization of each carbon source determined by ^13^C-NMR analysis of the culture supernatant. Both dividing and non-dividing promastigote stages displayed high rates of glucose uptake and overflow metabolism, with approximately 50% of the internalized glucose being secreted as succinate, acetate and alanine (derived from acetyl-CoA, pyruvate and PEP, respectively) (Fig. 1B, C). These promastigote stages also co-utilized glutamate (Fig. 1B) and a range of other non-essential amino acids, including aspartate, alanine, proline and glutamine [18] (unpublished data) which were also partially catabolized, contributing to the secretion of overflow metabolites (Fig. 1B). In contrast, Ama^axenic^ exhibited markedly lower rates of glucose and glutamate uptake (∼30-fold decrease in each case compared to Pro^log^). The decreased rate of utilization of these carbon sources was substantially greater than expected based on the ∼5-fold decrease in cell size of Ama^axenic^ compared to Pro^log^ (∼8 µm^3^ versus 40 µm^3^). Moreover, Ama^axenic^ exhibited undetectable levels of secretion of partially oxidized end-products (Fig. 1A,B and C), indicating complete oxidation of these carbon sources and/or utilization for biomass accumulation. Collectively, these findings indicate that, compared to both dividing and non-dividing promastigote stages, Ama^axenic^ exhibits a nutrient-sparing metabolism that is characterized by reduced uptake and more efficient catabolism of glucose and amino acids. We have termed this the stringent metabolic response.

To assess whether there was a correlation between the rate of nutrient uptake and parasite growth, Pro^log^, Pro^stat^ and Ama^axenic^ were suspended in medium containing 5% ^2^H_2_O and the rate of deuterium incorporation into the deoxyribose moiety of parasite DNA was determined by GC-MS. This method provides a highly sensitive and quantitative measure of DNA replication in slow-growing cells [24]. As expected, Pro^log^ DNA was rapidly labelled under these conditions consistent with a doubling time of 10 h, while labelling of Pro^stat^ DNA was negligible over several days, indicating growth arrest of the entire population (Fig. 1D). Ama^axenic^ exhibited a doubling time that was approximately 5-fold slower than that observed in Pro^log^ (Fig. 1D), consistent with estimates from other studies [25]. These analyses show that the development of the stringent glucose-sparing phenotype in Ama^axenic^ does not reflect entry of this stage into a growth-arrested state. Moreover, they indicate that growth arrest alone, as exhibited by Pro^stat^, does not lead to the stringent glucose-sparing phenotype. These analyses support the conclusion that the amastigote stringent metabolic response represents a distinct physiological state that is coupled to amastigote differentiation.

### Development of glucose-sparing metabolism in Ama^axenic^ is associated with changes in TCA cycle fluxes

**Figure 2:**
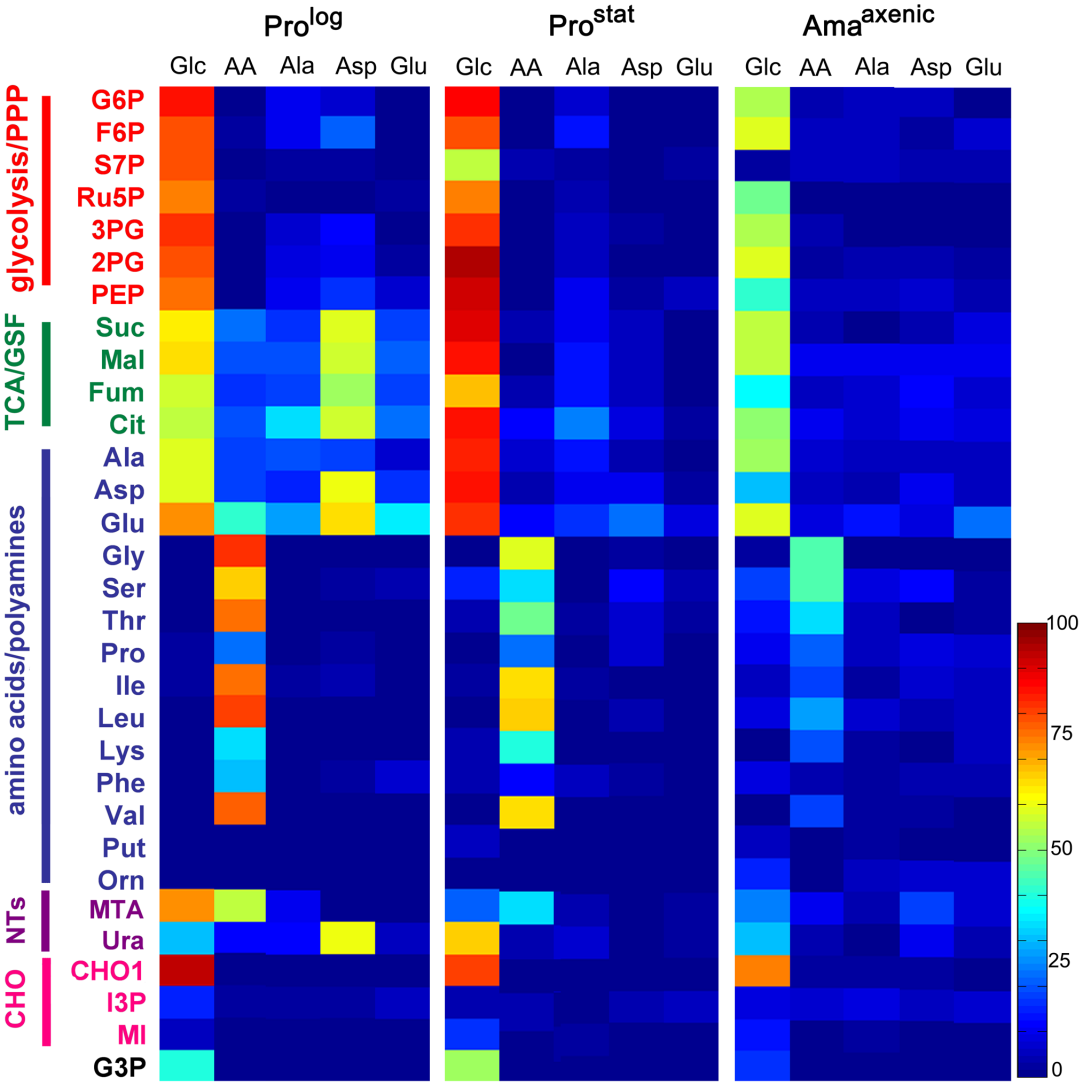
Isotopic enrichment of intracellular metabolite pools following cultivation with different ^13^C-labeled carbon sources. A. *L. mexicana* Pro^log^, Pro^stat^ and Ama^axenic^ were suspended in CDM containing either ^13^C-U-glucose (Glc), a mixture of ^13^C-U-amino acids (AA), ^13^C-U-alanine (Ala), ^13^C-U-aspartate (Asp), or ^13^C-U-glutamate (Glu) for 3 h and ^13^C-enrichment (mol percent) in intracellular intermediates in central carbon metabolism determined by GC-MS. Data shown for ^13^C-Glc and individual amino acid labeling in Pro^log^ panel was taken from [18]. Mean (n = 3) and SD for this data is provided in Tables S1–6 in Text S1. Abbreviations used are as follows G6P, glucose 6-phosphate; F6P, fructose 6-phosphate; S7P, seduheptulose 7-phosphate; Ru5P, ribulose 5-phosphate; 3PG, 3-phosphoglycerate; 2PG, 2-phosphoglycerate; PEP, phosphoenolpyruvate; Suc, succinate; Mal, malate; Fum, fumarate; Cit, citrate; Ala, alanine; Asp, aspartate; Glu, glutamate; Gly, glycine; Ser, serine; Thr, threonine; Pro, proline; Ile, isoleucine; Leu, leucine; Lys, lysine; Phe, phenylalanine; Val, valine; Put, putrescine; Orn, ornithine; MTA, 5-methylthioadenosine; Ura, uracil; CHO1, mannogen; I3P, inositol 3-phosphate; MI, *myo*-inositol; G3P, glycerol 3-phosphate.

To more precisely map carbon metabolism in different *L. mexicana* developmental stages, Pro^log^, Pro^Stat^ and Ama^axenic^ were cultivated in completely defined medium (CDM) containing different ^13^C-U-labelled carbon sources. Incorporation of label into 31 key intermediates, representative of different pathways in central carbon metabolism, was quantified by GC-MS (Fig. 2, Fig. S1 and Tables S1–S6 in Text S1). As previously shown, Pro^log^ co-catabolize ^13^C-U-glucose and ^13^C-U-aspartate and, to a lesser extent, ^13^C-U-alanine and ^13^C-U-glutamate (Fig. 2, Tables S1–S6 in Text S1) [18]. Label derived from ^13^C-U-glucose was incorporated into key intermediates of glycolysis, the pentose phosphate pathway (PPP), the glycosomal succinate fermentation (GSF) pathway and the TCA cycle (Fig. 2, Fig. S1 and Table S1–S2 in Text S1), consistent with previous models of metabolism [18]. Label was also incorporated into oligosaccharides derived from the major carbohydrate reserve material, mannogen, as well as into pyrimidine nucleotides and fatty acids (Fig. 2, Table S1 in Text S1). In contrast, label derived from aspartate, alanine and glutamate was almost entirely incorporated into TCA cycle intermediates with minimal assimilation into other pathways, indicating a high degree of metabolic compartmentalization of amino acid catabolism (Fig. 2, Fig. S2 and Tables S1–S6 in Text S1). While *Leishmania* can synthesize a number of amino acids *de novo*, including glycine, serine, threonine and proline [17], these amino acids were not labelled in these experiments (Fig. 2) indicating that they are primarily scavenged from the medium. This was confirmed by incubation of Pro^log^ with a cocktail of ^13^C-U-amino acids which were actively taken up, as demonstrated by strong labelling of corresponding intracellular amino acid pools (Fig. 2).

Consistent with the ^13^C-NMR analyses, Pro^stat^ also exhibited high rates of ^13^C-glucose uptake and incorporation of label into intermediates in glycolysis, PPP, GSF and the TCA cycle. Compared to Pro^log^, labelling of some Pro^stat^ PPP intermediates (including ribose-5-phosphate and seduheptulose-7-phosphate) was decreased, while labelling of TCA cycle intermediates increased (Fig. 2A), suggesting reduced demand for nucleotide synthesis and increased flux into the TCA cycle. Increased flux into the TCA cycle was particularly evident when levels of enrichment in TCA cycle intermediates were normalized relative to label in glucose-6-phosphate (Fig. S1). Uptake and catabolism of ^13^C-U-alanine, ^13^C-U-asparate and ^13^C-U-glutamate was also decreased in this stage compared to Pro^log^ (Fig. 2A), as was the labelling of intracellular pools of other amino acids when Pro^stat^ were provided with a cocktail of ^13^C-U-amino acids (Fig. 2A). This transition towards reduced nutrient uptake was further accentuated in Ama^axenic^. Labelling of all glycolytic, PPP, GSF pathway and TCA cycle intermediates in ^13^C-U-glucose-fed Ama^axenic^ were reduced compared to dividing or non-dividing promastigote stages after 3 h incubation (Fig. 2). This was not due to a switch to using other carbon sources, as the labelling of these intermediates with ^13^C-U-glucose was comparable when all three stages were labelled to isotopic steady-state over 24 h (data not shown). Interestingly, Ama^axenic^ appear to shut down the non-oxidative arm of the PPP, based on the markedly reduced labelling of seduheptulose 7-phosphate (Fig. 2, Fig. S1), possibly reflecting the need to use most of the ribose-5-phosphate generated by the oxidative-PPP for nucleotide synthesis and/or NADPH production under conditions of low glucose uptake.

**Figure 3:**
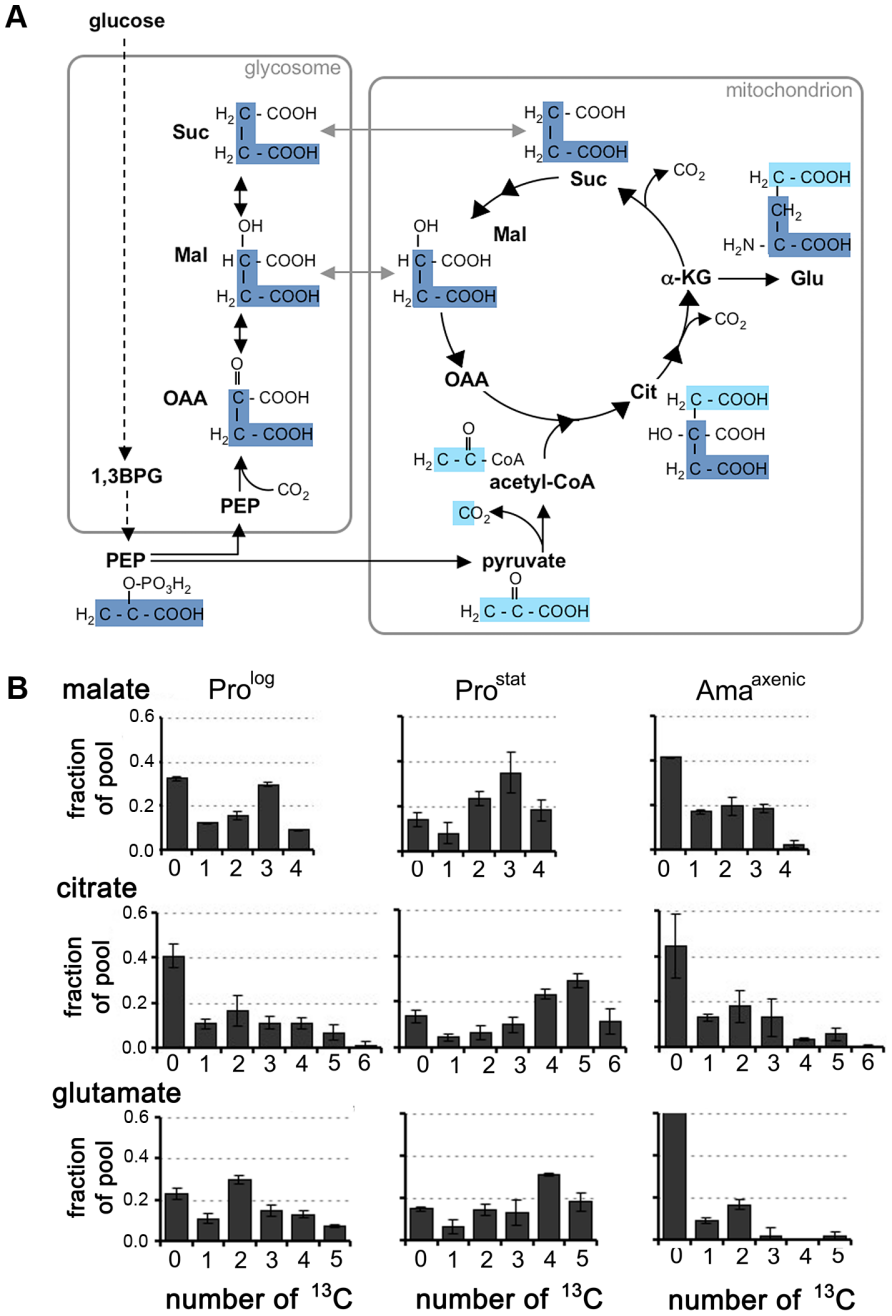
Stage-specific differences in TCA cycle fluxes. A. Scheme showing how label from ^13^C-U-glucose can be incorporated into TCA cycle intermediates. ^13^C_3-_PEP (carbon skeletons in dark blue) can be carboxylated in glycosomes to generate ^13^C_3_-labeled OAA, malate, and succinate. These C4 dicarboxylic acids are used to top up the TCA cycle depleted during glutamate synthesis. Alternatively, PEP can be directly catabolized to pyruvate (carbon skeletons in light blue) and ^13^C-U-acetyl-CoA. B. Mass isotopomers of malate, citrate and glutamate in *L. mexicana* Pro^log^, Pro^Stat^ and Ama^axenic^ labeled with ^13^C-U-glucose for 3 h. These data are taken from the labeling experiment shown in Fig. 2, and are representative of several other experiments. Data represent mean (n = 3) and SD.

Our labelling studies suggested that all developmental stages catabolize glucose via both the GSF pathway and the mitochondrial TCA cycle. This was confirmed by analysis of the mass isotopomer distribution of these metabolites (Fig. 3A). Specifically, the presence of a prominent malate +3 mass isotopomer in all stages is consistent with operation of the GSF pathway in which ^13^C_3_-PEP is converted to ^13^C_3_-oxaloacetate by the glycosomal PEP carboxykinase [18]. The ^13^C_3_-oxaloacetate is subsequently converted to ^13^C_3_-malate and ^13^C_3_-succinate in the glycosome. Conversely, the presence of M+1 malate isotopomer may reflect the incorporation of a single labelled carbon from ^13^CO_2_ generated by other enzymes, such as pyruvate dehydrogenase and the TCA cycle. The dicarboxylic acids generated by the GSF are further catabolized in the mitochondrial TCA cycle as shown by the presence of significant levels of M+3 citrate isotopomers in all stages. The carbon backbones of hexose also enter the TCA cycle as ^13^C-U-acetyl-CoA, as demonstrated by the presence of M+2 and M+4 citrate isotopomers (derived from entry of one or two C2 units, respectively) and M+5 citrate isotopomers (derived from entry of ^13^C_3_-dicarboxylic acids and ^13^C_2_-acetyl-CoA). The presence of other citrate isotopomers (including M+1 and M+6) are consistent with intermediates participating in multiple TCA cycles.

Stage-specific differences were observed in the isotopomer labelling patterns of specific metabolites, providing additional information on stage-specific changes in metabolic fluxes. Specifically, there was a pronounced shift towards labelling of M+4, M+5 and M+6 citrate isotopomers in ^13^C-glucose-fed Pro^stat^ compared to Pro^log^ or Ama^axenic^ (Fig. 3B), consistent with TCA enzymes operating in a highly cyclic manner to generate reducing equivalents. In contrast, the citrate pool in Pro^log^ and Ama^axenic^ contained predominantly M+2 and M+3 isotopomers and negligible levels of fully labelled M+6 citrate, consistent with operation of the TCA cycle in anabolic mode in which newly synthesized intermediates are siphoned off for synthesis of other metabolites. In particular, the export α-ketoglutarate for glutamate synthesis in both Pro^log^ and Ama^axenic^ was suggested by the presence of predominant M+2 glutamate isotopomers in these stages (Fig. 3B). Overall, these labelling studies suggest that Pro^stat^ primarily generate ATP from oxidative phosphorylation, while Pro^log^ and Ama^axenic^ appear to utilize a compartmentalized TCA cycle in which carbon skeletons derived from glucose primarily enter the TCA cycle as malate or acetyl-CoA providing α-ketoglutarate for the synthesis of glutamate/glutamine.

### Fatty acid β-oxidation is upregulated in Ama^axenic^

**Figure 4:**
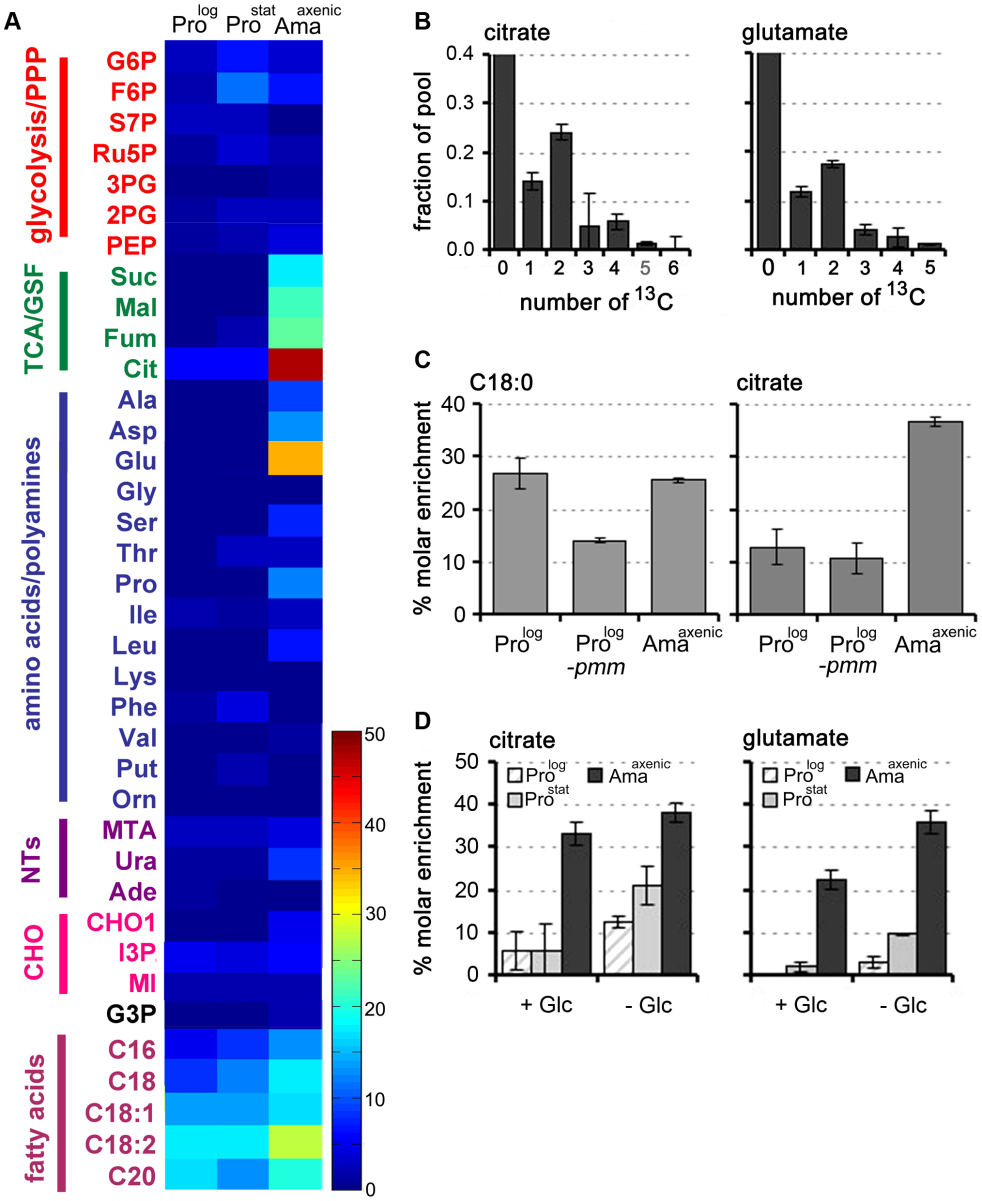
Fatty acid β-oxidation is increased in *L. mexicana* Ama^axenic^. A. *L. mexicana* promastigote and amastigote stages were suspended in CDM supplemented with ^13^C-U-fatty acids for 3 h and isotopic enrichment (mol percent) in intermediates of central carbon metabolism was determined by GC-MS. Abbreviations as for Figure 2. This data (mean (n = 3) and SD) is also presented in Tables S7 in Text S1. B. Mass isotopomers of citrate and glutamate in ^13^C-U-fatty acid fed (3 h) *L. mexicana* Ama^axenic^. C. Wild type (WT) Pro^log^ and Ama^axenic^ and glycocalyx deficient Δ*pmm*-Pro^log^ parasites were labeled with ^13^C-U-fatty acids for 48 h and isotopic enrichment in the total lipid fraction (exemplified by hexadecanoic acid, C18:0) and citrate was determined by GC-MS. D. Fatty acid β-oxidation is elevated during glucose starvation. *L. mexicana* Pro^log^, Pro^stat^ and Ama^axenic^ were labeled with ^13^C-U-fatty acids for 3 h in glucose-replete (+Glc) or glucose-free (−Glc) CDM and isotopic enrichment in intracellular pools of citrate and glutamate determined by GC-MS. Data represent mean (n = 3) and SD.

While *L. mexicana* Pro^log^ can take up free ^13^C-U-fatty acids, these are not normally catabolized to acetyl-CoA by β-oxidation under standard culture conditions [6]. Fatty acid β-oxidation is also repressed in Pro^stat^ under glucose-replete conditions, as shown by the absence of detectable labelling of TCA cycle intermediates in ^13^C-U-fatty acid labelled parasites (Fig. 4A, Table S7). In contrast, incubation of Ama^axenic^ with ^13^C-U-fatty acids resulted in appreciable labelling of TCA cycle intermediates even in the presence of glucose (Fig. 4A). High levels of ^13^C-enrichment were observed in citrate and α-ketoglutarate/glutamate, while other TCA cycle intermediates and inter-connected metabolites, such as aspartate, were labelled to a lesser extent. The major isotopomers of citrate and glutamate labelled under these conditions contained two ^13^C atoms, consistent with entry of ^13^C_2_-acetyl-CoA from β-oxidation of fatty acids (Fig. 4B).

The increased fatty acid β-oxidation in Ama^axenic^ could reflect more efficient uptake and/or internalization of fatty acids as a consequence of this stage lacking a conspicuous surface glycocalyx [26]. To investigate this possibility, we examined the rate of uptake of ^13^C-U-fatty acid and β-oxidation in *L. mexicana *Δ*pmm* promastigotes, which are unable to synthesize the major surface GPI-anchored proteins and glycolipids and lack a surface glycocalyx [27]. Rates of ^13^C-fatty acid uptake and β-oxidation were not increased in *L. mexicana *Δ*pmm* Pro^log^ compared to wild type Pro^log^ parasites (Fig. 4C) indicating that the glycocalyx does not impede the intercalation of fatty acids into the plasma membrane. In contrast, fatty acid β-oxidation was induced when WT Pro^log^ and Pro^stat^ were suspended in glucose-free medium (Fig. 4D). Fatty acid β-oxidation in Ama^axenic^ was also elevated under glucose starvation conditions, although the increase was less prominent than in the promastigote stages. These analyses indicate that fatty acid β-oxidation is activated in promastigotes in response to glucose starvation, but is constitutively active in Ama^axenic^, most likely reflecting the down-regulation of glucose uptake in this stage.

### Lesion-derived amastigotes exhibit a similar metabolic remodelling

**Figure 5:**
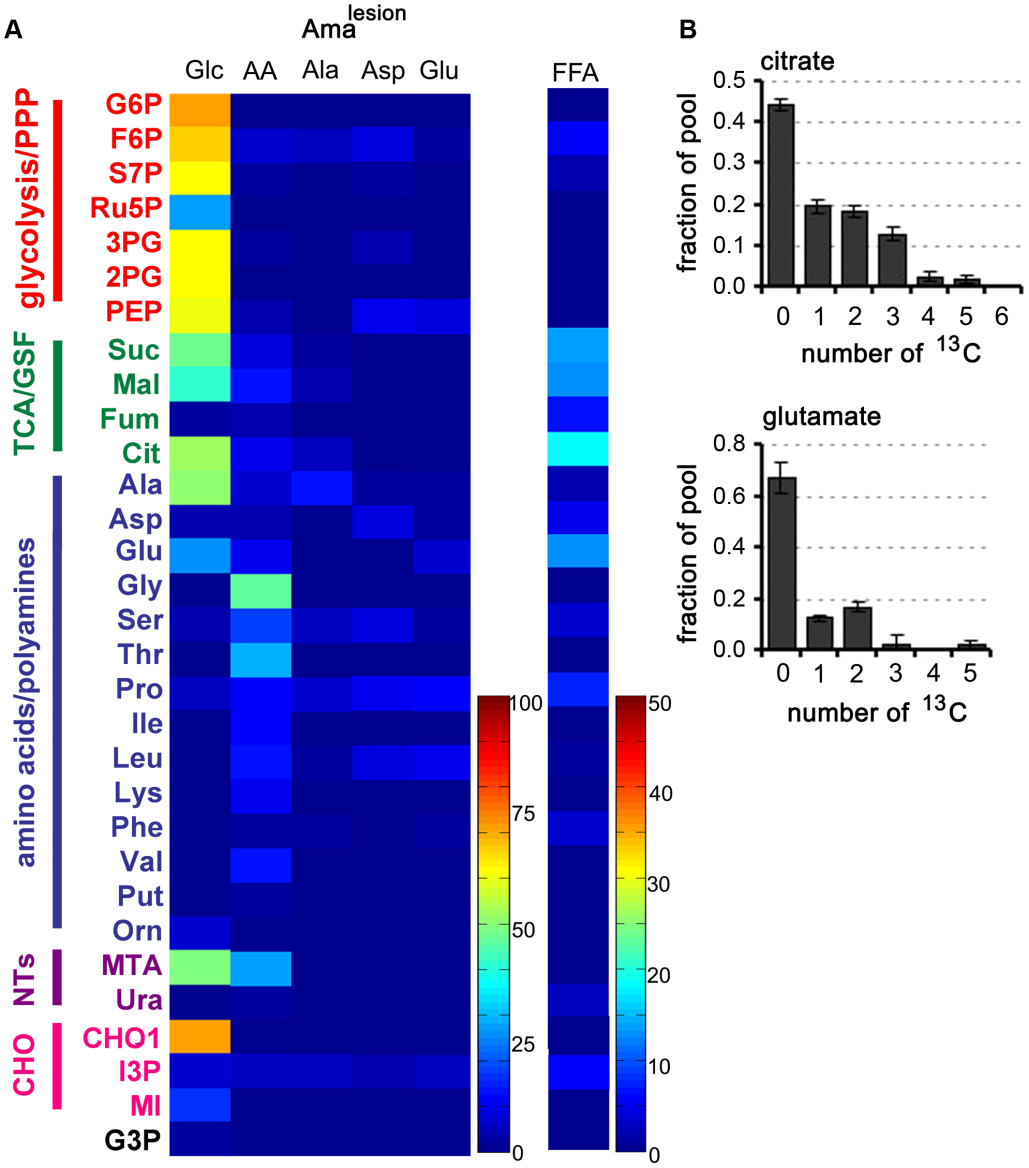
Carbon source utilization by Ama^lesion^. A. *L. mexicana* amastigotes were harvested from susceptible Balb/c mice lesions and purified Ama^lesion^ immediately suspended in CDM containing either ^13^C-U-glucose (Glc), a mixture of ^13^C-U-amino acids (AA), ^13^C-U-alanine (Ala), ^13^C-U-aspartate (Asp), ^13^C-U-glutamate (Glu), or ^13^C-U- fatty acids (FFA). Isotopic enrichment in intracellular pools of metabolites in central carbon metabolism was determined by GC-MS (see also Tables S1–S7 in Text S1). B. The uptake of ^13^C-U-glucose and the secretion of labeled end products in Ama^lesion^ were determined by ^13^C-NMR analysis of culture supernantant after 24 h incubation. C. Mass isotopomer of citrate and glutamate in Ama^lesion^ labelled with ^13^C-U-fatty acids for 3 h. Data represent mean (n = 3) and SD.

We next investigated whether amastigotes isolated from mouse lesions (Ama^lesion^) exhibit the same metabolic phenotype as Ama^axenic^. Ama^lesion^ were purified from BALB/c mouse lesions and immediately suspended in the medium containing different ^13^C-U-labelled carbon sources (Fig. 5A, Tables S1–S7 in Text S1). In common with Ama^axenic^, Ama^lesion^ exhibited low rates of ^13^C-U-glucose uptake and secretion of organic acids compared to the promastigote stages (Fig. 5A, unpublished data). ^13^C-U-glucose was incorporated into intermediates in the glycolytic and non-oxidative PPP, while labelling of ribose-5P, the end-product of the oxidative PPP was low (Fig. 5A, Fig. S1). Similar to the situation in Ama^axenic^, TCA cycle intermediates and glutamate were enriched with +2 and +3 isotopomers indicating limited TCA cycling (Fig. 5B,C) and cataplerotic export of TCA cycle intermediates for glutamate synthesis. The uptake of ^13^C-labeled alanine, aspartate and glutamate, as well as the cocktail of essential amino acids, was similarly reduced indicating a general down-regulation of nutrient transport systems (Fig. 5A). Finally, Ama^lesion^ also displayed elevated levels of fatty acid β-oxidation with incorporation of C2 units into both citrate and glutamate (Fig. 5A, unpublished data).

### Operation of a TCA cycle is required for Ama^axenic^ viability

**Figure 6:**
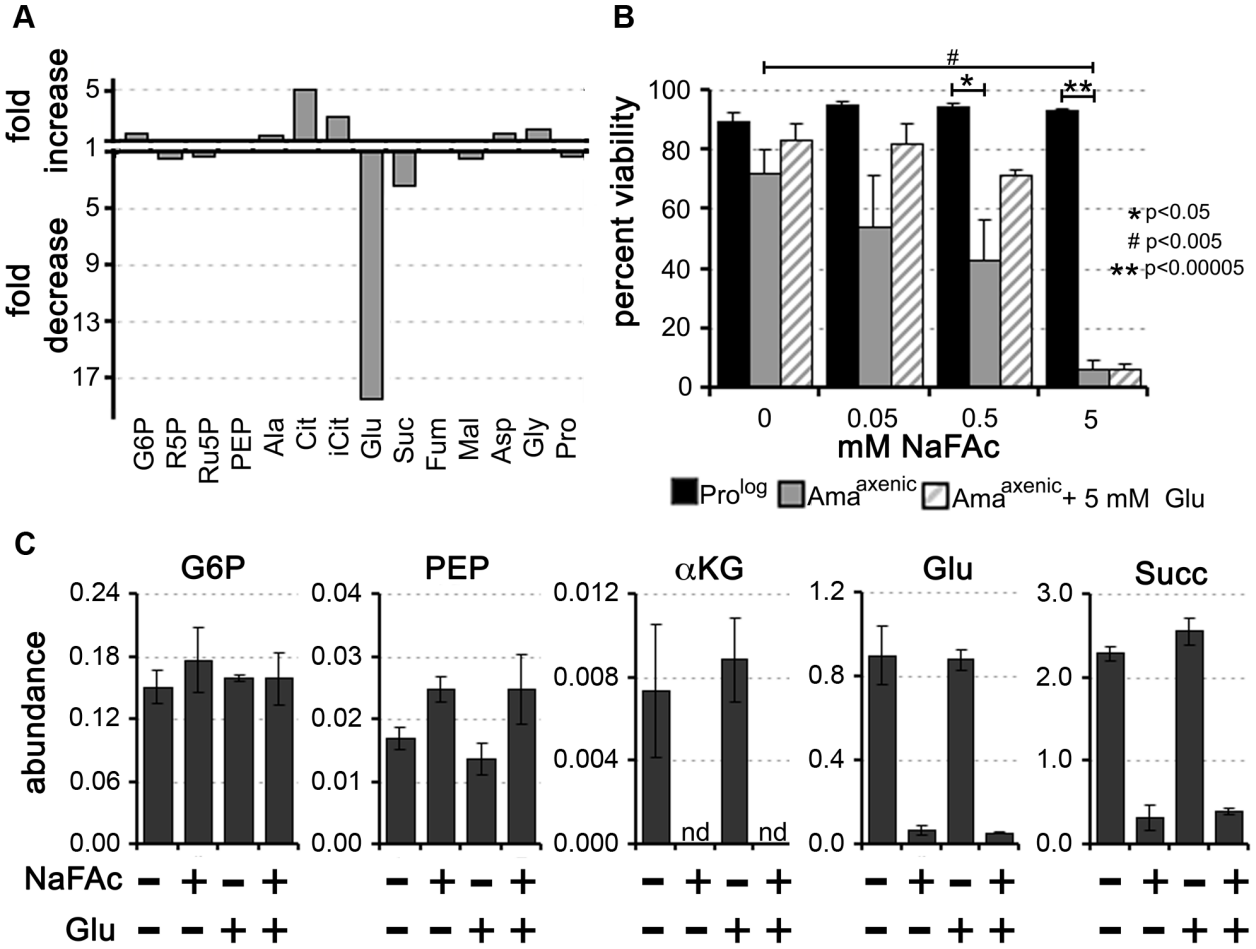
Inhibition of the TCA cycle in Ama^axenic^ is cytotoxic. Sodium fluoroacetate (NaFAc) results in inhibition of the aconitase reaction in the TCA cycle. A. Ama^axenic^ were treated with 0.5 mM NaFAc and the steady-state levels of selected intermediates in central carbon metabolism determined by GC-MS. The fold-change in NaFAc-treated compared to sodium acetate-treated Ama^axenic^ are shown. For abbreviations see Figure 2 and R5P, ribose 5-phosphate and iCit, isocitrate. B. Pro^log^ and Ama^axenic^ were cultivated in RPMI medium containing 10% iFCS supplemented with NaFAc (0.05 to 5 mM) for 48 h and viability assessed by labeling with propidium iodide. NaFAc toxicity in Ama^axenic^ was partially reversed by supplementation of the medium with 5 mM glutamate (background level of glutamate in the medium is 0.15 mM). Data represented as mean (n = 3–4) +/− SEM, *, *P<0.05*; **, *P<0.00005* and #, *P<0.005* C. Intracellular levels of key Ama^axenic^ metabolites following incubation in the presence or absence of NaFAc or glutamate. Exogenous glutamate did not lead to restoration of intracellular glutamate levels following NaFAc treatment. Data represent the mean (n = 3) and SD.

Our labelling studies suggested that Ama^axenic^ and Ama^lesion^ utilize a compartmentalized TCA cycle in which C4 dicarboxylic acids and acetyl-CoA, derived from glucose and fatty acids, are converted to citrate and glutamate. The conversion of citrate to α-ketoglutarate is dependent on the enzyme aconitase, which can be selectively inhibited with sodium fluoroacetate (NaFAc) following its metabolic conversion to fluoroacetyl-CoA and fluorocitrate [28]. NaFAc treatment results in effective inhibition of *L. mexicana* Pro^log^ aconitase and reversible growth arrest of this stage [18]. Similarly, treatment of Ama^axenic^ with NaFAc led to the selective inhibition of the TCA cycle, as shown by the accumulation of citrate and isocitrate and concomitant decrease in intracellular pools of glutamate (Fig. 6A, Table S8 in Text S1). Intriguingly, while extended exposure (48 h) of Pro^log^ to 5 mM NaFAc had not effect on viability, treatment of Ama^axenic^ with a 100-fold lower concentration of NaFAc was cytotoxic, resulting in dose-dependent loss of viability (Fig. 6B). Cytotoxicity at low NaFAc concentrations (0.05–0.5 mM) was partially rescued by supplementation of the Ama^axenic^ culture medium with 5 mM glutamate (Fig. 6B). Significantly, intracellular pools of glutamate remained low in NaFAc-treated Ama^axenic^, even in the presence of 1 mM exogenous glutamate, suggesting that glutamate uptake is insufficient to sustain normal intracellular levels (Fig. 6C). Addition of glutamate to the medium did not rescue cytotoxicity at 5 mM NaFAc, possibly due to secondary toxicity caused by NaFAc-induced accumulation of citrate [28]. Together, these results support the conclusion that Ama^axenic^ have a reduced capacity to scavenge glutamate from the medium, even when intracellular pools are depleted and are therefore dependent on the anabolic functions of the TCA for *de novo* synthesis of this amino acid.

### Intracellular amastigotes are dependent on an active TCA cycle for glutamate/glutamine synthesis

**Figure 7:**
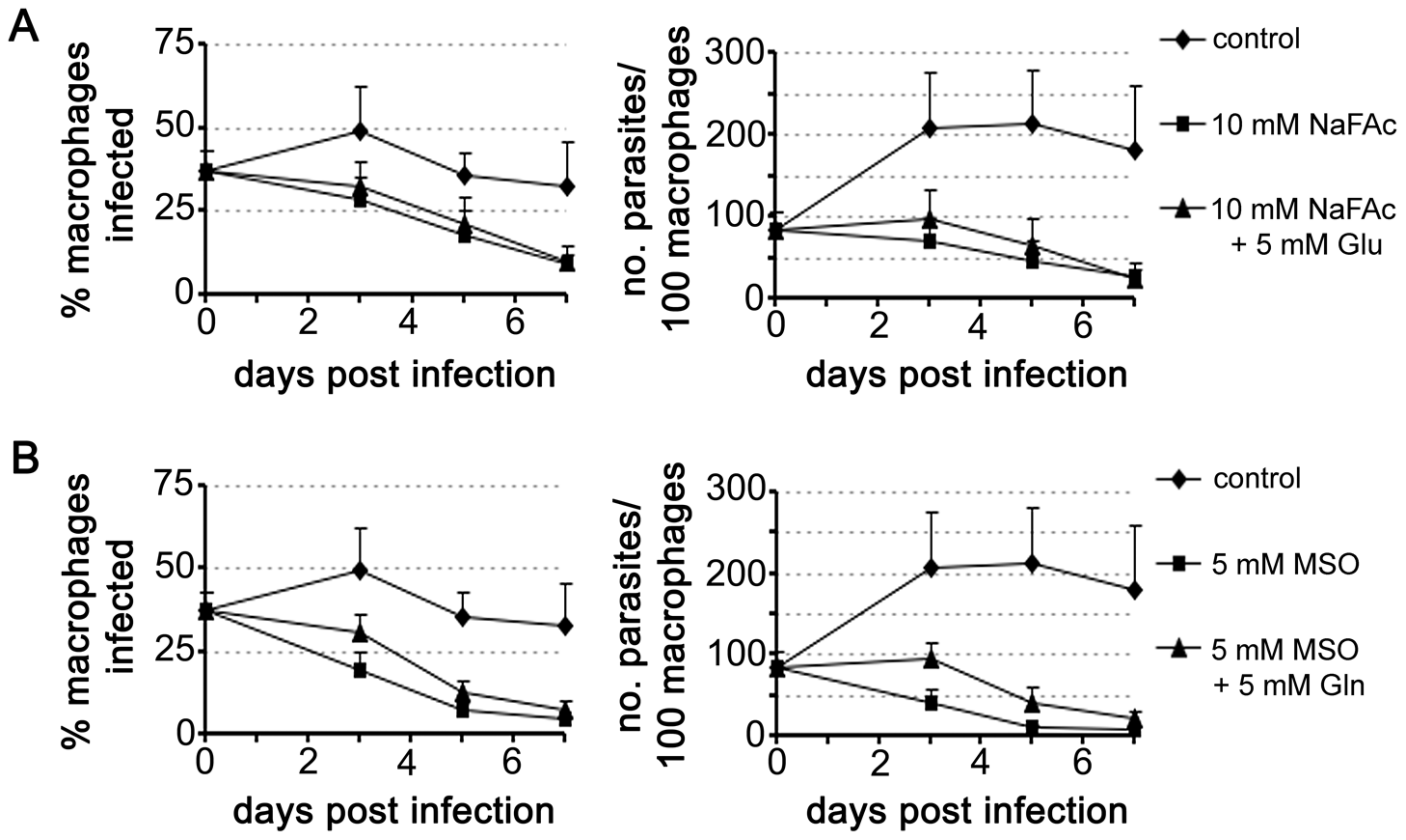
Intracellular *L. mexicana* amastigotes are dependent on TCA cycle function and glutamate/glutamine synthesis. RAW 264.7 macrophages were infected with *L. mexicana* Ama^axenic^ for 4 h and then incubated in RPMI supplemented with the metabolic inhibitors (A) sodium fluoroacetate (NaFAc, 10 mM) or (B) methionine sulphoximine (MSO, 5 mM). The medium of infected macrophages was also supplemented with either Glu or Gln (5 mM), as indicated. Percent infection and number of parasites per macrophage were determined by microscopy. Data represent the mean (n = 3) and SEM.

To determine whether amastigotes are dependent on an active TCA cycle while growing in macrophages, *L. mexicana* infected RAW264.7 macrophages were treated with NaFAc (Fig. 7A). RAW264.7 macrophages are highly glycolytic and addition of NaFAc has no detectable effect on the long-term growth or viability of these host cells (data not shown). However, NaFAc treatment led to a progressive decrease in both the parasite load and the number of amastigote-infected macrophages. Addition of extra glutamate (5 mM) to medium to by-pass any potential changes in host cell metabolism, did not rescue amastigote growth (Fig. 7A), suggesting that NaFAc treatment has a direct effect on parasite growth rather than inhibiting parasite growth indirectly through changes in macrophage metabolism.

Glutamate is converted to glutamine, the amino donor for several essential metabolic pathways in *Leishmania*, including pyrimidine and amino sugar synthesis [29], [30]. This reaction is catalyzed by glutamine synthetase, which enables the ATP-dependent amidation of the γ-carboxyl group of glutamate. Chemical inhibition of this enzyme in Pro^log^ with methionine sulfoximine (MSO) results in a dose-dependent growth arrest that can be by-passed with addition of exogenous glutamine (Fig. S2). MSO-treatment of *L. mexicana* infected macrophages led to the effective inhibition of intracellular amastigote proliferation (Fig. 7B). MSO had no effect on the growth or viability of uninfected macrophages (data not shown). Furthermore, supplementation of the medium with glutamine, which would by-pass any glutamine deficiency in the macrophage caused by MSO treatment, did not promote intracellular amastigote growth. Together, these results suggest that intracellular amastigote stages are dependent on the early steps of the TCA cycle for the *de novo* synthesis of glutamate and/or glutamine.

## Discussion

*Leishmania* amastigotes proliferate long-term within the mature phagolysosomal compartment of mammalian macrophages. How they survive within this hostile niche remains largely undefined. Here, we show that amastigote differentiation is associated with decreased growth rate and acquisition of a nutrient-sparing metabolic state. These changes are replicated in lesion-derived amastigotes and are likely to represent an important adaptive response to growth within the mammalian host.

**Figure 8:**
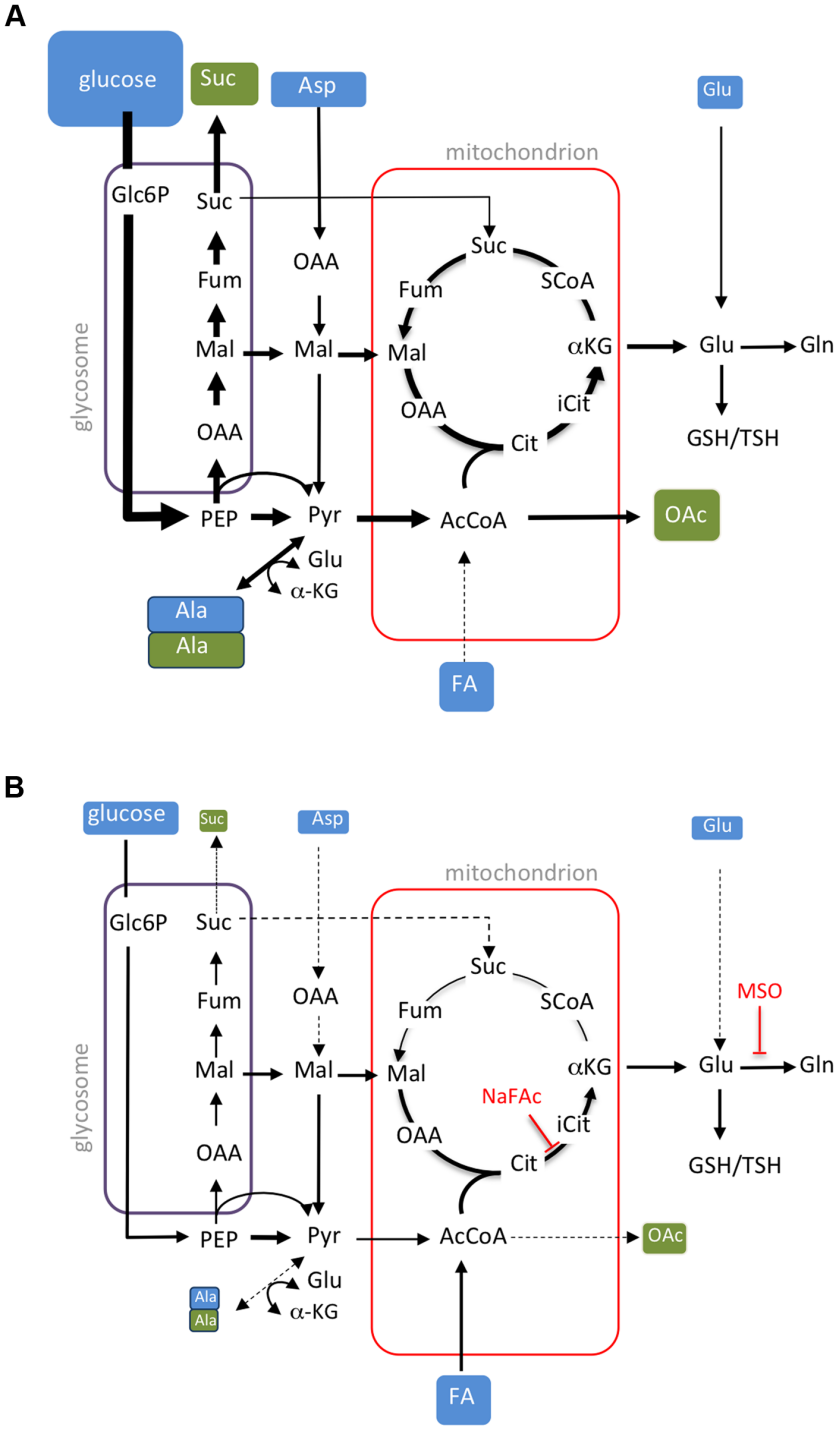
Remodelling of *L. mexicana* central carbon metabolism during amastigote differentiation. Key pathways of carbon utilization in *L. mexicana* promastigotes (A) and amastigotes (B) are represented. Major carbon sources (blue box) and overflow metabolites (open box) detected in this study are shown. Fluxes through dotted pathways are down-regulated relative to the other stage. Steps inhibited by NaFAc and MSO are indicated. Abbreviations used, αKG, α-ketoglutarate; AcCoA, acetyl-CoA; Ala, alanine; Asp, aspartate; Cit, citrate; Fum, fumarate; FA, fatty acids; G6P, glucose 6-phosphate; G3P, glyceraldehyde 3-phosphate; Gln, glutamine; Glu, glutamate; Mal, malate; OAA, oxaloacetate; OAc, acetate; PEP, phosphoenolpyruvate; PPP, pentose phosphate pathway; Pro, proline; Pyr, pyruvate; SCoA, succinyl-CoA; Suc, succinate; TCA, tricarboxylic acid cycle.

Previous studies have shown that *Leishmania* promastigotes preferentially catabolize glucose via glycolysis, succinate fermentation and a full TCA cycle [18], [20], [31]. Under standard culture conditions, rapidly dividing promastigotes utilize more glucose than they need, resulting in a high rate of overflow metabolism and secretion of partially oxidized end-products, such as succinate, acetate and alanine [18]. Non-dividing promastigotes were found to exhibit a similar glucose-wasting phenotype, despite having lower rates of glucose consumption compared to Pro^log^, indicating that major pathways of carbon metabolism in these stages are regulated down-stream of glucose uptake. In striking contrast, Ama^axenic^ and Ama^lesion^ develop a glucose-sparing metabolism characterized by greatly reduced rates of glucose consumption and negligible secretion of metabolic end-products (Fig. 8B). We have termed this switch to a glucose-sparing phenotype the stringent metabolic response. Our findings are consistent with previous reports showing that amastigotes exhibit low rates of glucose uptake [20]. However, we show here that glucose uptake is not linked to an increase in amino acid catabolism and only to a limited increase in fatty acid β-oxidation. In fact the rate of uptake and catabolism of a range of non-essential and essential amino acids pools was dramatically decreased in Ama^axenic^ and Ama^lesion^, suggesting that the stringent metabolic response is coupled to a global decrease in protein and lipid biosynthesis, a conclusion supported by other recent studies [12], [32]. We have previously shown that Ama^axenic^ and Ama^lesion^ also accumulate high levels (>10 mM) of the intracellular carbohydrate reserve material, mannogen [33]. This material was rapidly labelled in all developmental stages (Fig. 1), raising the possibility that mannogen turnover has a role in regulating hexose-phosphate levels and flux into glycolysis and the PPP. Taken together, these findings indicate that amastigotes establish a unique nutrient-sparing metabolic state that is linked to more efficient energy metabolism, a decrease in energy intensive cellular processes, such as protein and lipid biosynthesis, and the redirection of carbon into intracellular carbohydrate reserves.

Ama^axenic^ and Ama^lesion^ exhibited broadly similar rates of glucose, amino acid and fatty acid utilization, indicating that the development of a stringent metabolic response is coupled to differentiation signals rather than nutrient depletion *per se*. Specifically, the Ama^axenic^ develop this response despite being suspended in rich medium (supplemented with fresh fetal calf serum) that contains high levels of glucose and amino acids. Similarly, Ama^lesion^ exhibited the same stringent metabolic phenotype following their transfer to glucose-rich medium after isolation. Finally, extended cultivation of Pro^stat^ in identical medium to Ama^axenic^ in the absence of increased temperature or reduced pH, did not lead to the glucose sparing phenotype (data not shown). These findings suggest that the amastigote stringent metabolic response is primarily induced by differentiation signals such as elevated temperature and/or reduced pH, although the possibility that depletion of micronutrients also contributes to the establishment of this response cannot be discounted. Regardless of the signal, amastigote differentiation appears to involve hard-wired changes in nutrient uptake and metabolic fluxes that are coupled to growth restriction.

Consistent with previous studies [10], [11], [20], we show that *L. mexicana* Ama^axenic^ and Ama^lesion^ have increased rates of fatty acid β-oxidation. However, *Leishmania* are unable to use fatty acids as their sole carbon source [5], [17] and the fate of the acetyl-CoA generated by fatty acid β-oxidation has not been previously investigated. Here, we show that ^13^C-U-fatty acids are co-catabolized with glucose in a partially compartmentalized TCA cycle that is primarily directed towards the biosynthesis of glutamate/glutamine. C2 units derived from fatty acids are combined with C4 dicarboxylic acids generated by the glycosomal succinate fermentation pathway to synthesize citrate which is subsequently converted to α-ketoglutarate and glutamate (Fig. 8B). As carbon skeletons derived from glucose can also enter the TCA cycle as acetyl-CoA, the primary function of fatty acid β-oxidation may be to spare PEP from entering the TCA cycle via pyruvate dehydrogenase. PEP spared by this process can be converted to oxaloacetate by the carbon-fixing PEP carboxykinase, increasing carbon yield and metabolic efficiency, as well as contributing to the maintenance of the redox/energy balance of the glycosomes (Fig. 8B). Fatty acid catabolism may thus allow glucose catabolism to be uncoupled from production of NADH and allow fine control of both the redox balance and production of essential anabolic precursors in the mitochondrion. These findings explain why *Leishmania* mutants with defects in hexose uptake, hexosamine catabolism and fatty acid β-oxidation exhibit very strong loss of virulence phenotypes in susceptible animal models [21], [23], [34], and indicate that enzymes in these pathways of carbon utilization are potential drug targets.

Our data suggest that *Leishmania* amastigotes are highly dependent on mitochondrial metabolism for *de novo* synthesis of glutamate and glutamine. Amastigotes maintain significant intracellular pools of these amino acids which are required for amino acid homeostasis as well as the synthesis of the major cellular thiols (glutathione/trypanothione), pyrimidines and amino sugars, that are all individually essential for parasite growth and stress responses [29], [30], [35]. Chemical inhibition of the mitochondrial aconitase or glutamine synthetase in intracellular amastigotes led to parasite clearance indicating that these stages are unable to salvage sufficient glutamate or glutamine from the macrophage phagolysosome. Intriguingly, Ama^axenic^ were also found to be acutely sensitive to NaFAc treatment *in vitro*. In contrast to promastigote stages, which cease growing but remain viable when exposed to high concentrations of NaFAc [18], Ama^axenic^ lose viability at 10 to 100-fold lower concentrations of NaFAc, and cytotoxicity is only partially reversed by exogenous glutamate. The acute sensitivity of cultivated and intracellular amastigotes to NaFAc treatment correlated with the reduced capacity of this stage to take up exogenous glutamate or other amino acids, such as glutamine or proline, that could be converted to glutamate. It remains to be determined why amastigote stages down-regulate glutamate and glutamine transport. The phagolysosome compartment is one of the major sites of protein degradation in macrophages and is thought to contain relatively high level of free amino acids [5]. High rates of amino acid uptake and catabolism could lead to increased TCA cycle flux and reductive stress, as a result of excessive production of reducing equivalents and superoxide generation by the mitochondrial respiratory chain [36], [37]. The repression of glutamate uptake in amastigotes may therefore represent an adaptive strategy to minimize TCA flux and oxidative stress. Regardless of the underlying basis, the dependence of amastigotes on the TCA cycle for glutamate synthesis would contribute to their dependence on hexose and fatty acid catabolism and may underlie the increased sensitivity of this stage to genetic or chemical inhibition of other mitochondrial functions such as the respiratory chain and oxidative phosphorylation [38], [39]. However, while continuous cycling around the TCA cycle in amastigotes was limited, oxidative phosphorylation may still be required to maintain cellular ATP levels and critical functions such as neutral intracellular pH, under conditions of low glucose flux.

How these stage-specific changes in *Leishmania* metabolism are regulated in the absence of gene-specific transcriptional regulation remains poorly defined [40]. We have recently shown that the major *L. mexicana* hexose transporters are internalized and degraded in the parasite lysosome in response to heat shock and amastigote differentiation [41]. Nutrient transporter internalization may be regulated by the mono-ubiquitination of the cytoplasmic domains of these proteins [41] and coupled with a global decrease in protein synthesis [12], [32]. The decreased surface expression of hexose transporters and possibly other nutrient transporters [42] is likely to play a key role in generating the amastigote stringent metabolic phenotype. In contrast to glucose uptake, we show that promastigotes and amastigotes constitutively take up fatty acids and that fatty acid β-oxidation is stimulated under glucose limiting conditions in promastigotes or as a result of the stage-specific down-regulation of glucose catabolism in amastigotes (this study, [43]). Thus the regulation of fatty acid β-oxidation may occur primarily at the post-translational or metabolic level [34].

While there is little information on the growth rate of *Leishmania* amastigotes in infected animals, our data suggest that the maximum growth rate of amastigotes is ∼5 fold slower than that of promastigotes (Fig. 1D). A slow growth rate is likely to be advantageous for amastigote development in the nutrient-limited phagolysosome compartment of macrophages. There is also abundant evidence that entry into a slow growth, metabolically quiescent state is associated with increased resistance to a variety of stresses, including elevated temperature and oxidative stress in bacteria and other single-celled eukaryotes [44]–[47]. Intriguing, the metabolism of *Leishmania* amastigotes exhibits some similarities with other microbial pathogens, such as *Mycobacterium tuberculosis* that, like *Leishmania*, can also establish long-term latent infections in humans or other animal host. In particular, slow growing stages of *M. tuberculosis* appear to be dependent on the co-utilization of both sugars and fatty acids (or other lipids) as carbon sources and utilize anaplerotic pathways to uncouple glucose oxidation from production of reducing equivalents [46], [48], [49]. The minimization of metabolically induced redox stress may therefore be a common adaptation for pathogens that live in host cells with active oxidative defences such as macrophages.

In summary, we show that *Leishmania* amastigote differentiation is associated with programmed entry into nutrient sparing, stringent metabolic state. We propose that this growth-limiting state facilitates amastigote survival within the nutrient-limited macrophage phagolysosome, while paradoxically increasing the dependency of this stage on hexose uptake and catabolism. These studies have focussed on the metabolism of lesion-derived amastigotes harvested during acute stages of infection and open the way for similar studies on the physiological state of amastigotes during the early stages of infection as well as during long term chronic infections.

## Materials and Methods

### Ethics statement

Use of mice was approved by the Institutional Animal Care and Use Committee of the University of Melbourne (ethics number 0811011.1). All animal experiments were performed in accordance with the Australian National Health Medical Research council (Australian code of practice for the care and use of animals for scientific purposes, 7^th^ Edition, 2004, ISBN: 1864962658).

### Parasite strains and growth conditions

*L. mexicana* wild type (WT) (MNYC/BZ/62/M379) promastigotes were cultured in RPMI 1640 medium (Sigma) supplemented with 10% heat inactivated foetal calf serum (iFCS, GibcoBRL) at 27°C. Δ*pmm* parasites [27] were cultured in M199 medium supplemented with 10% iFBS. Parasites were passaged twice weekly (1/100 and 1/1000) into fresh media to maintain log phase growth. Promastigotes were harvested at mid-log (Pro^log^) and stationary (Pro^stat^) growth phases (day 2 and 5 after inoculum, respectively). Pro^stat^ were induced to differentiate to axenic amastigotes (Ama^axenic^) using a variation of the method described by Bates et al [50]. Briefly, Pro^Stat^ cultures were acidified to pH 5.5 by addition of HCl, supplemented with an additional 10% iFBS and then incubated at 33°C. Amastigote-like forms were visible by day three and mature Ama^axenic^ were harvested at day five. For inhibitor studies, Pro^log^ and Ama^axenic^ were incubated with sodium fluoroacetate (NaFAc, 0–5 mM) for 48 h, with or without additional glutamate supplementation (5 mM over background concentration of 0.25 mM). Parasite viability was determined by staining the cells with propidium iodide (0.2 µg/ml in PBS, 5 min, on ice) and non-viable parasites quantified by microscopy (Axioplan 2 fluorescence imaging microscope, Carl Zeiss).

### BALB/c mouse inoculation and harvesting of intracellular lesion-derived amastigotes

Female BALB/c mice (6–8 weeks), derived from a specific pathogen-free facility, were inoculated subcutaneously at the base of the tail with Pro^stat^ (10^6^ parasite/50 µl PBS). Dissected lesions were initially suspended in RPMI 1640 medium supplemented with 10% iFBS (for transport) and Ama^lesion^ immediately extracted by differential filtration as previously described [51], with modifications. The tissue was initially passed through a fine sieve and infected macrophages lyzed by syringe passage through a 27 G needle. Host debris was removed by centrifugation (50× *g*, 5 min) and the resulting supernatant recentrifuged (805× g, 10 min) to pellet the released Ama^lesion^. The purity (RBC- and macrophage-free) of the final Ama^lesion^ preparation was determined by light microscopy (×100).

### ^13^C-stable isotope metabolic labelling experiments

^13^C-stable isotope labelling experiments were performed as described previously [18]. Briefly, individual developmental stages were suspended in chemically defined media (CDM) [52] containing various uniformly ^13^C-labeled carbon sources at a density of 2×10^7^ cell/ml. CDM containing the following ^13^C-U-labeled carbon sources (Cambridge Stable Isotopes) were used: ^13^C-U-glucose (6 mM), ^13^C-U-alanine (1.9 mM), ^13^C-U-aspartate (1.5 mM), ^13^C-U-glutamine (1.5 mM), ^13^C-U-glutamate (1.5 mM), and ^13^C-U-fatty acids. ^13^C-U-fatty acids were conjugated to delipidated bovine serum albumin (BSA, 1∶1 mole/mole) and the fatty acid-BSA conjugate added to CDM at 0.5% (w/v).

### ^2^H_2_O labelling of DNA

Pro^log^, Pro^stat^ and Ama^axenic^ were cultured in media supplemented with PBS containing 100% D_2_O to give a final concentration of 5% ^2^H_2_O (v/v). DNA extracted from parasites at selected time points [53] was hydrolyzed and dephosphorylated as described in [54]. The released deoxyribose moiety (250 µl) was oximated by addition of 0.01 M HCl (1.84 ml) and O-(2,3,4,5,6-pentafluorobenzyl) hydroxylamine acetate (20 µl, 25 mg/ml) and incubated (90°C, 3 h) [55]. Oximes were extracted in 2 steps using an ethyl acetate/hexane mix (1∶1 v/v) and pure ethyl acetate. Pooled organic phases were dried under a stream of nitrogen and deoxyribose silylated by addition of ethyl acetate (10 µl) and TMS reagent (N,O-bis(trimethylsilyl) trifluoroacetamide containing 1% trimethylchlorosilane, Pierce, 20 µl) at 90°C for 30 min. Samples were analysed by GC-MS with a DB5 capillary column (J&W Scientific, 30 m, 250 µm i.d., 0.25 µm film thickness, containing a 10 m inert duraguard) [18]. Derivatized sugars were detected in negative chemical ionization (NCI) mode with methane as reagent gas. Sugar peaks were identified based on GC-retention times and mass spectra in comparison to authentic standards, using MSD ChemStation Data Analysis Application (Agilent). Individual ions were extracted and enrichment was determined from the ratio of monoisotopic molecular ions and the +1 mass isotopomer, after correction for occurrence of natural isotopes of carbon, nitrogen, hydrogen, oxygen, and silicon in both the metabolite and derivatizing agent as described previously [18], [56].

### Extraction and analysis of small metabolites

Pro^log^, Pro^stat^, Ama^axenic^ and Ama^lesion^ metabolism was quenched by rapid chilling as previously described [18]. Briefly, triplicate aliquots of parasite suspensions (12×10^7^ parasites) were transferred to 15 ml tubes, immersed in a dry ice-ethanol slurry (∼10 seconds to cool the cell suspension to 0°C), and then transferred to an ice-water slurry. The chilled parasites were centrifuged (805× *g*, 10 min, 0°C) and the culture supernatant removed and stored at −70°C for analysis. Parasite pellets was suspended in ice-cold PBS (4×10^7^ cell equivalents, 3 ml) and washed twice with cold PBS (10,000× *g*, 1 min, 0°C) and immediately extracted in chloroform:methanol:water (CHCl_3_:CH_3_OH:H_2_O, 1∶3∶1 v/v, 250 µl) containing 1 nmol *scyllo*-inositol as internal standard. The extracts were vortex mixed and then incubated at 60°C for 15 min. Insoluble material was removed by centrifugation (16,100× *g*, 0°C, 5 min) and the supernatant adjusted to CHCl_3_:CH_3_OH:H_2_O (1∶3∶3 v/v) by addition of H_2_O, and vortex mixed and centrifuged (16,000× *g*, 5 min) to induce phase separation. The lower phase was retained for lipid analysis while the upper phase was transferred to a fresh microfuge tube (1.5 ml) for analysis of polar metabolites. Polar phases were dried *in vacuo* (55°C) in 250 µl glass vial inserts and free aldehyde groups protected by derivatization in methoxyamine chloride (Sigma, 20 µl, 20 mg/ml in pyridine) with continuous mixing (14 h, 25°C). Metabolites were derivatized with TMS reagent (N,O-bis(trimethylsilyl) trifluoroacetamide containing 1% trimethylchlorosilane (Pierce, 20 µl, 1 h, 25°C) and analysed by GC-MS [18]. Apolar lipid phases (2×10^7^ cell equivalents) were subjected to solvolysis in methanolic HCl prior to derivatisation with *N*-Trimethylsilyl-*N*-methyl trifluoroacetamide (MSTFA) containing 1% trimethylchlorosilane (TMCS) and analysed by GC-MS [18]. Metabolites were identified with reference to authentic standards (retention time and spectra), were possible, and in conjunction with MSD ChemStation Data Analysis Application (Agilent) using in-house and Wiley metabolite libraries. All metabolites, presented in heat maps, were corrected for background labelling as described above, and the level of labeling shown as the percent of the metabolite pool containing one or more ^13^C atoms [18]. Heat maps were created in R (http://www.r-project.org/). All other graphs and statistical analyses were preformed in Microsoft Office Excel.

### Analysis of carbon source utilization and over-flow metabolites by ^13^C-NMR

Parasites were labelled for 24 h, and the cell-free culture supernatant (540 µl) gently premixed with 5 mM D_6_-DSS in D_2_O (60 µl, containing 0.2% w/v NaN_3_), 21.4 mM ^13^C-U-glycerol in D_2_O (5 µl, containing 0.2% w/v NaN_3_) and 21.4 mM imidazole in D_2_O (5 µl, containing 0.2% w/v NaN_3_), prior to analysis by NMR. ^13^C-spectra at 200 MHz were obtained using a 800 MHz Bruker-Biospin Avance fitted with a cryoprobe [18]. Quantification was performed as previously published [57] and spectra assigned by reference to authentic standards.

### Macrophage infections

RAW 264.7 macrophages were plated onto glass coverslips (24 well plate) in RPMI supplemented with 10% iFBS and allowed to adhere overnight (37°C, 5% CO_2_). Monolayers were overlaid with Ama^axenic^ at a MOI of 5 (4 h, 33–34°C, 5% CO_2_) and then washed three times with PBS before being overlaid with fresh RPMI medium containing 10% iFBS supplemented with varying concentrations of NaFAc (0–10 mM) or methionine sulfoxime (0–5 mM). Infected macrophages were incubated for 3–7 days (33–34°C, 5% CO_2_) and adherent cells washed with PBS and then fixed with chilled methanol (5 min). Fixed cells were stained with hoechst (8 µg/ml in Mowiol) and host and parasite nuclei visualized by fluorescence microscopy.

## Supporting Information

Figure S1 **Normalization of ^13^C-glucose labeling data to glucose-6-phosphate.** The level of labeling of indicated metabolites following incubation of Pro^log^, Pro^Stat^, Ama^axenic^ and Ama^lesion^ with ^13^C-U-glucose for 3 h was normalized to label in glucose-6-phosphate (100%) to highlight relative changes in some metabolic fluxes (Data also tabulated in Table S7 in Text S1).
(TIF)

Figure S2 **The glutamine synthetase inhibitor, methionine sulfoximine, causes *L. mexicana* promastigotes to growth arrest which is reversed in the presence of exogenous glutamine.** A. *L. mexicana* Pro^log^ were cultivated in glutamine-free M199 medium containing 10% iFCS and methionine sulfoximine (MSO, 0.1 to 5 mM) and parasite growth monitored by change in OD_600_. B. *L. mexicana* Pro^log^ were cultivated in M199 medium with or without MSO and/or glutamine. Promastigote growth in the presence of MSO was restored by supplementation of the medium with 0.685 mM glutamine. All data represent mean (n = 3) and SEM. * *P<0.05*.
(TIF)

Text S1 **Supporting tables.**
(DOC)
