# Supplementary figures and images for: Induction of a Stringent Metabolic Response in Intracellular Stages of Leishmania mexicana Leads to Increased Dependence on Mitochondrial Metabolism
Source: PLoS Pathog. 2014 Jan 23;10(1):e1003888. doi: 10.1371/journal.ppat.1003888 (PMC3900632; doi:10.1371/journal.ppat.1003888)

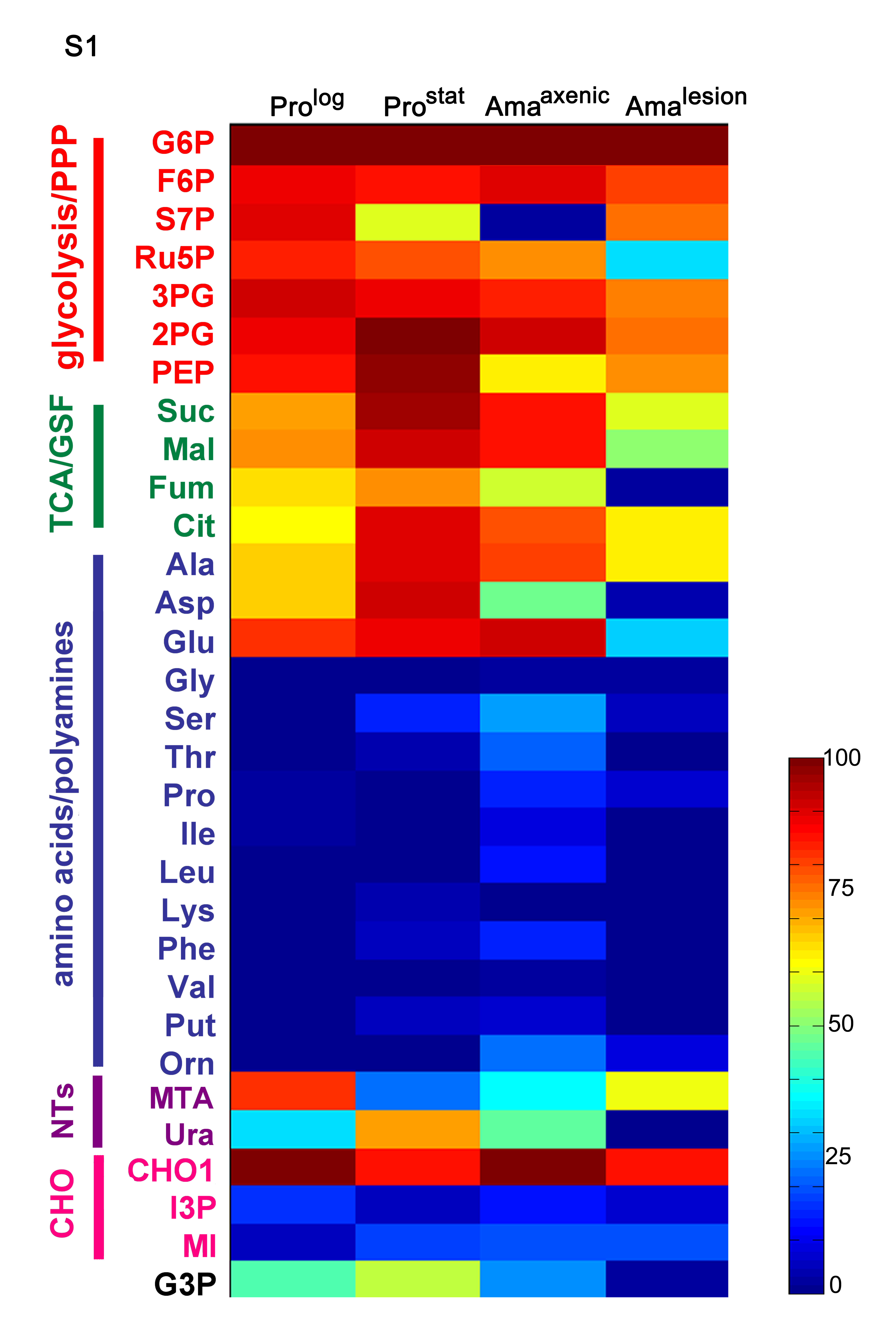

Supplement: Figure S1 — Normalization of 13C-glucose labeling data to glucose-6-phosphate. The level of labeling of indicated metabolites following incubation of Prolog, ProStat, Amaaxenic and Amalesion with 13C-U-glucose for 3 h was normalized to label in glucose-6-phosphate (100%) to highlight relative changes in some metabolic fluxes (Data also tabulated in Table S7 in Text S1). (TIF) [file ppat.1003888.s001.tif]

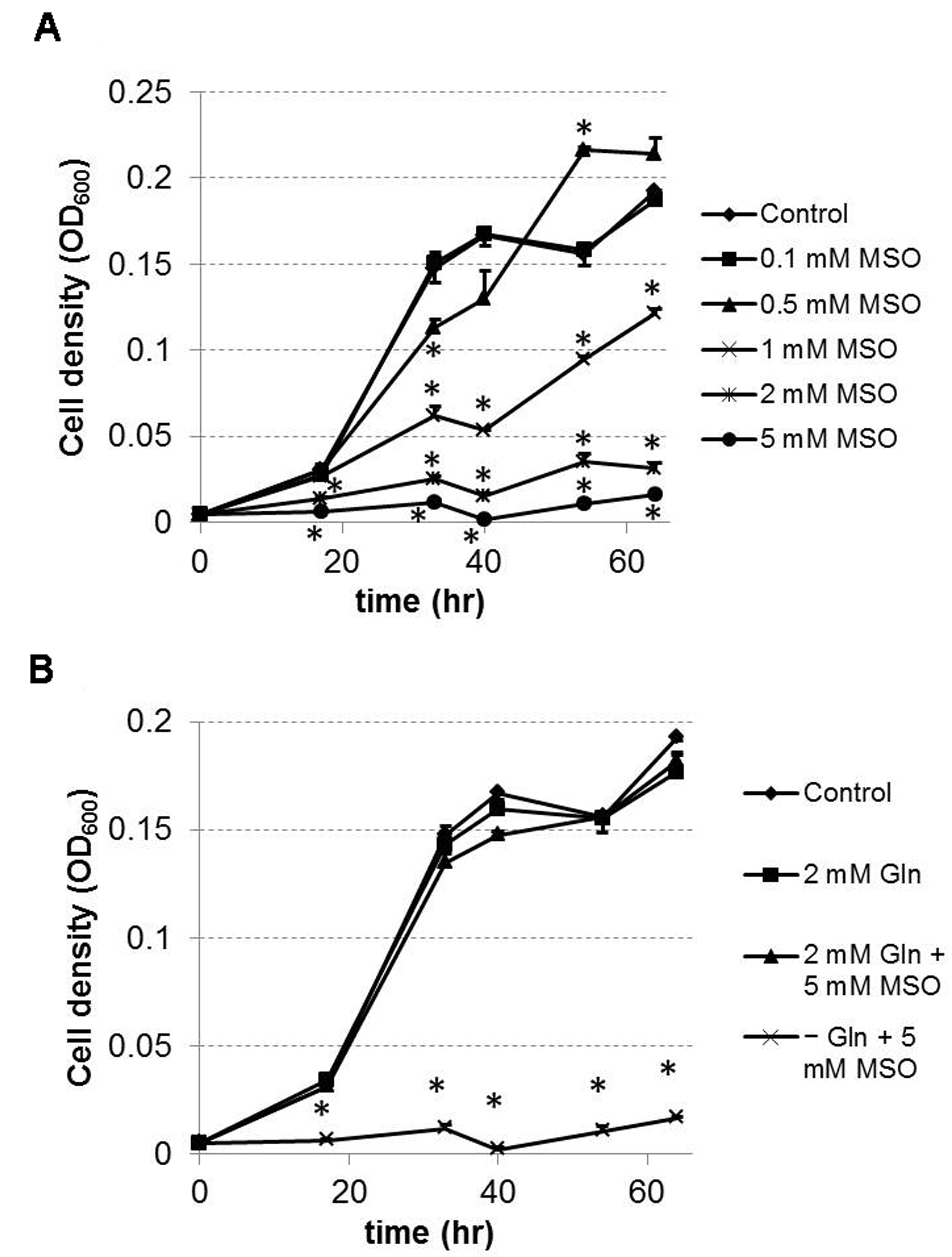

Supplement: Figure S2 — The glutamine synthetase inhibitor, methionine sulfoximine, causes L. mexicana promastigotes to growth arrest which is reversed in the presence of exogenous glutamine. A. L. mexicana Prolog were cultivated in glutamine-free M199 medium containing 10% iFCS and methionine sulfoximine (MSO, 0.1 to 5 mM) and parasite growth monitored by change in OD600. B. L. mexicana Prolog were cultivated in M199 medium with or without MSO and/or glutamine. Promastigote growth in the presence of MSO was restored by supplementation of the medium with 0.685 mM glutamine. All data represent mean (n = 3) and SEM. * P<0.05. (TIF) [file ppat.1003888.s002.tif]
