# Supplementary material for: Induction of a Stringent Metabolic Response in Intracellular Stages of Leishmania mexicana Leads to Increased Dependence on Mitochondrial Metabolism
Source: PLoS Pathog. 2014 Jan 23;10(1):e1003888. doi: 10.1371/journal.ppat.1003888 (PMC3900632; doi:10.1371/journal.ppat.1003888)
Supplement: Text S1 — Supporting tables. (DOC) [file ppat.1003888.s003.doc]

**Table S1. *L. mexicana* developmental stages labelled with ^13^C-U-glucose.** *L. mexicana* Pro^log^ (MLP), Pro^stat^ (SP), Ama^axenic^ (AA) and Ama^lesion^(LA) were suspended in CDM containing ^13^C-U-glucose for 3 h. ^13^C-enrichment (as mol percent) in intracellular intermediates in central carbon metabolism determined by GC-MS. Diagnostic ions used calculate ^13^C-enrichment are indicted ('Ion selected').

Abbreviations used: G6P, glucose 6-phosphate; F6P, fructose 6-phosphate; S7P, seduheptulose 7-phosphate; Ru5P, ribulose 5-phosphate; 3PG, 3-phosphoglycerate; 2PG, 2-phosphoglycerate; PEP, phosphoenolpyruvate; Suc, succinate; Mal, malate; Fum, fumarate; Cit, citrate; Ala, alanine; Asp, aspartate; Glu, glutamate; Gly, glycine; Ser, serine; Thr, threonine; Pro, proline; Ile, isoleucine; Leu, leucine; Lys, lysine; Phe, phenylalanine; Val, valine; Put, putrescine; Orn, ornithine; MTA, 5-methylthioadenosine; Ura, uracil; CHO1, mannogen; I3P, inositol 3-phosphate; MI, *myo*-inositol; G3P, glycerol 3-phosphate; C16; hexadecanoic acid; C18: octadecanoic acid: C18:1: Octadecacenoic acid: C18:2: Octadecadienoic acid; C20: Eicosanoic acid.

**Table S2 *L. mexicana* developmental stages labelled with ^13^C-U-glucose - scaled to G6P.** *L. mexicana* Pro^log^ (MLP), Pro^stat^ (SP), Ama^axenic^ (AA) and Ama^lesion^(LA) were suspended in CDM containing ^13^C-U-glucose for 3 h. ^13^C-enrichment (as mol percent) in intracellular polar metabolites determined by GC-MS. Enrichment was scaled to G6P (471 m/z ion) (see also Figure S1)

**Table S3. *L. mexicana* developmental stages labelled with ^13^C-U-alanine.** *L. mexicana* Pro^log^ (MLP), Pro^stat^ (SP), Ama^axenic^ (AA) and Ama^lesion^(LA) were suspended in CDM containing ^13^C-U-alanine for 3 h. ^13^C-enrichment (as mol percent) in intracellular polar metabolites was determined by GC/MS

**Table S4. *L. mexicana* developmental stages labelled with ^13^C-U-aspartate.** *L. mexicana* Pro^log^ (MLP), Pro^stat^ (SP), Ama^axenic^ (AA) and Ama^lesion^(LA) were suspended in CDM containing ^13^C-U-aspartate for 3 h. ^13^C-enrichment (as mol percent) in intracellular polar metabolites was determined by GC-MS.

**Table S5. *L. mexicana* developmental stages labelled with ^13^C-U-glutamate.** *L. mexicana* Pro^log^ (MLP), Pro^stat^ (SP), Ama^axenic^ (AA) and Ama^lesion^(LA) were suspended in CDM containing ^13^C-U-glutamate for 3 h. ^13^C-enrichment (as mol percent) in intracellular polar metabolites was determined by GC-MS.

**Table S6**. ***L. mexicana* developmental stages labelled with ^13^C-U-mixed amino acids.** *L. mexicana* Pro^log^ (MLP), Pro^stat^ (SP), Ama^axenic^ (AA) and Ama^lesion^(LA) were suspended in CDM containing ^13^C-U-mixed amino acids for 3 h. ^13^C-enrichment (as mol percent) in intracellular intermediates in central carbon metabolism determined by GC-MS.

**Table S7**. ***L. mexicana* developmental stages labelled with ^13^C-U-free fatty acids.** *L. mexicana* Pro^log^ (MLP), Pro^stat^ (SP), Ama^axenic^ (AA) and Ama^lesion^(LA) were suspended in CDM containing ^13^C-U-FFA for 3 h. ^13^C-enrichment (as mol percent) in intracellular polar metabolites was determined by GC-MS.

**Table S8**. **Sodium fluoroacetate (NaFAc) results in inhibition of the aconitase reaction in the TCA cycle.** Ama^axenic^ were treated with 0.5 mM NaFAc in CDM supplemented with ^13^C-U-FFA. Relative abundance and labelling (as mol percent) of selected intermediates in central carbon metabolism were determined by GC-MS. For abbreviations see Figure S1 and R5P, ribose 5-phosphate and iCit, isocitrate. See figure 6 for fold-change.
